# Effects of ERK/p38 MAPKs signaling pathways on MTA-mediated osteo/odontogenic differentiation of stem cells from apical papilla: a vitro study

**DOI:** 10.1186/s12903-020-1016-x

**Published:** 2020-02-12

**Authors:** Jing Du, Yating Lu, Mengxiao Song, Lin Yang, Junqing Liu, Xinyu Chen, Yue Ma, Yan Wang

**Affiliations:** 1 Department of VIP center, School and Hospital of Stomatology, Shandong University & Shandong Key Laboratory of Oral Tissue Regeneration & Shandong Engineering Laboratory for Dental Materials and Oral Tissue Regeneration, No. 44-1 Wenhua Road West, Jinan, 250012 Shandong China; 2 Department of Stomatology, Liaocheng People’s Hospital, Liaocheng, China; 3 Department of Stomatology, The First Affiliated Hospital of Zhengzhou University & Department of Oral Pathology, School of Stomatology, Zhengzhou University, No. 40 University Road, Zhengzhou, 450052 Henan China; 4 Department of Endodontics, Jinan Stomatological Hospital, Jinan, 250001 China

**Keywords:** Stem cells from apical papilla, Mineral trioxide aggregate, Osteo/odontogenic differentiation, MAPKs signaling pathways

## Abstract

**Background:**

Stem cells from apical papilla (SCAP) located in the root apex of immature permanent teeth are a reliable cell source for pulp-dentine complex regeneration. Mineral trioxide aggregate (MTA) is a biocompatible material which has been widely used in endodontic treatments. The aim of this study was to elucidate the regulatory role of MTA in the proliferation and differentiation of SCAP.

**Methods:**

Cell viability was detected by Cell counting kit-8. Characteristics of SCAP were confirmed by Flow cytometric (FCM) analysis and alizarin red staining. Then, MTA-mediated osteo/odontogenic differentiation of SCAP was investigated by reverse transcription polymerase chain reaction. The effect of MAPKs on MTA-mediated osteo/odontogenic differentiation was evaluated by western blot analysis.

**Results:**

There was no significant difference in cell viability between the control group and the group with lower concentrations of MTA. However, higher concentrations of MTA could inhibit proliferation of SCAP. It is demonstrated that the ALP activity were enhanced, the mRNA and protein expression of BSP, OCN, DSPP, Runx2 were up-regulated. In addition, phosphorylation proteins of ERK, p38 were activated through western blot analysis.

**Conclusions:**

MTA at appropriate concentration could enhance osteo/odontogenic differentiation of SCAP by activating p38 and ERK signaling pathways. This study provides a new idea for the clinical application of MTA and the treatment of endodontic diseases.

## Background

Root development and apical closure in permanent teeth require three or more years after eruption. Trauma or caries during this period usually leads to pulp necrosis and periapical periodontitis. Subsequently, the root development is stagnant with fragile root canal walls and open apices [1]. Previous reports showed that even the immature permanent teeth which clinically diagnosed with periapical periodontitis could undergo continual maturation of the root and apexogenesis [2]. The histologic and cell origin of the root development is a pivotal problem in immature permanent teeth.

Mesenchymal stem cells acquired from different tissues play an important role in various life activities [3–5]. The osteo/odontogenic differentiation of mesenchymal stem cells is regulated by networks composed of numerous signaling molecules, transcription factors and receptors, such as bone morphogenetic protein (BMP) and basic fibroblast growth factor (bFGF). Usually, we choose osteogenic differentiation medium to induce osteo/odontogenic differentiation of mesenchymal stem cells. Due to the sound stemness properties and differentiation potential, dental mesenchymal stem cells have become a research focus in regenerative medicine [6–8]. As a kind of dental Mesenchymal stem cells, stem cells from apical papilla (SCAP) were found and cultured by Sonoyama et al. for the first time [9]. The SCAP are located in the root apex of immature permanent teeth which have multiple differentiation potential and can be induced into osteo/odontoblasts, lipoblasts and neuroblasts in vitro. Compared with dental pulp stem cells, SCAP showed a superior potential of proliferation and osteogenic differentiation [10, 11]. In a developing minipig model, both SCAP and periodontal ligament stem cells (PDLSCs) with their scaffold were transplanted into the lower incisor socket, then, dentine and PDL tissue were regenerated [12]. In another study, Huang et al. inserted the SCAP/scaffolds into root fragments and implanted them into subcutaneous space of immunodeficient mice, the regenerated dentin-like tissue was formed after 3 months [13]. These studied showed that SCAP might be a promising cell source for pulp-dentine complex regeneration.

Recently, many studies have explored the effect of materials on osteogenic differentiation of mesenchymal stem cells and its related mechanism [14–16]. Mineral trioxide aggregate (MTA) is a biocompatible material and widely used in endodontic treatments, such as pulp capping, pulpotomy, perforation repair and apexification [17, 18]. With a good sealing ability, MTA protects teeth from microleakage better than traditional materials [19, 20]. MTA enhances the formation of reparative dentin bridge at exposed pulp after pulp capping and perforation repair [21]. Previous studies showed that MTA could accelerate the odontogenic differentiation of DPSCs in vitro [22]. In clinical reports, MTA promotes dental hard tissue formation in apexification of immature permanent teeth [18, 23]. Whereas, few researches are available to elucidate how MTA influences the biological behaviors of SCAP.

Mitogen-activated protein kinases (MAPKs) are serine/threonine-specific protein kinases that play an important role in cellular biological activities, including proliferation, differentiation, and apoptosis by transducing extracellular stimuli into cells. MAPKs include 3 families: extracellular signal-regulated kinases (ERKs), c-Jun N-terminal kinases (JNKs), and p38 MAPK [24]. Many factors are involving in the differentiation of Mesenchymal stem cells via MAPK signaling pathways [25, 26]. Previous study has shown that MTA could activate MAPK signaling pathways to promote osteo/odontogenic differentiation of dental pulp cells in vitro [27]. However, it is not explicit whether MAPKs signaling pathways participate in MTA-mediated osteo/odontogenic differentiation of SCAP.

In this study, we investigated the effect of MTA on proliferation and osteo/odontogenic differentiation in SCAP, meanwhile, the role of MAPKs signaling pathways in this process was explored. These findings revealed that MTA could regulate the osteo/odontogenic differentiation of SCAP via MAPK signaling pathways.

## Methods

### Cell culture

Human third permanent molars with immature root were obtained from the patients (14 to 18 years old) who undergone tooth extraction for orthodontic treatment at Stomatological Hospital of Shandong University. The apical papilla was separated carefully from the root apex and cut into pieces, then digested in a solution composed of collagenase type I (3 mg/mL) (Sigma-Aldrich Co, St. Louis, MO, USA) and dispase (4 mg/mL) (Worthington Biochemicals Corp, NJ, USA) for 30 min at 37 °C. The cells were cultured with alpha-Modification of Eagle’s Medium (α-MEM; Thermo scientific, Waltham, MA, USA) supplemented with 15% fetal bovine serum (FBS; Sijiqing, Hangzhou, China), 100 U/ml penicillin and 100 mg/ml streptomycin (Gibco, Grand Island, NY, USA) at 37 °C in 5% CO_2_. When the cells reached 80% confluence, they were passaged with the ratio 1:3. The cells at passages 2–5 were used in the following experiments.

### Flow cytometric (FCM) analysis

To further identify the cultured cell characteristics, isolated cells were tested using a flow cytometric analysis with specific surface antigens. STRO-1 and CD146 are the surface markers of mesenchymal stem cells. CD24 is a specific marker for SCAP, and CD45 appears to be a surface marker for hematopoietic precursors. The passage 2 SCAP were used in this assay, cell suspensions were harvested and incubated in dark with the following fluorchrome-conjugated rabbit anti-human antibodies: STRO-1-FITC, CD146-PE, CD24-FITC and CD45-FITC (BD Biosciences, San Jose, CA, USA) at 4 °C for 30 min. Then, the cells were washed twice in PBS and analyzed by flow cytometry. The experiment was repeated in triplicate.

### Osteogenic/adipogenic differentiation induction

Cells were seeded in 6-well plates at a density of 1 × 10^5^ /well. When reaching 60% confluence, the cells were treated with serum-free α-MEM for 24 h, then they were cultured in osteo/odontogenic differentiation medium supplemented with 10% FBS, 50 mg/ml ascorbic acid, 10 nM dexamethasone and 10 mM β-glycerophosphate (Sigma-Aldrich Co, St. Louis, MO, USA) or in OriCell Human Mesenchymal Stem Cell Adipogenic Differentiation Medium (Cyagen Biosciences, Guangzhou, China). After 4wk, Alizarin Red and Oil Red O staining were performed to visualize the mineralized nodules and lipid droplets.

### Cell counting kit-8

The SCAP were seeded in 96-well plates at 5000/well in α-MEM containing 10% FBS. Then, the serum-free medium was replaced until 60% confluence. After 24 h, cells were cultured with fresh medium containing different concentrations of MTA. The concentrations were 20, 10, 2, 0.2, 0.02 and 0.002 mg/ml, based on previous reports [9]. Each concentration included 3 replicate wells and a control well. The cell proliferation rate was analyzed using cell counting kit-8 (CCK-8; BestBio, Shanghai, China) on 1, 3, 5, and 7d. The cell proliferation rate was measured at a wavelength of 450 nm by microplate reader.

### Alkaline phosphatase activity

The SCAP were seeded in 6-well plates at a density of 1 × 10^5^ cells per well. When the cells reached 60% confluence, the medium was replaced by serum-free α-MEM. After 24 h, the cells were cultured in α-MEM with 10% FBS and different concentrations of MTA (0.02, 0.2 and 2 mg/ml). The medium without MTA was used as a control. The ALP activity was measured after 3 and 5 d using an ALP kit (Nanjing Jiancheng Technological Inc., Nanjing, China) according to the manufacturer’s protocol. The absorbance values at 520 nm were quantified using a microplate reader.

### Quantitative real-time polymerase chain reaction assays (qRT-PCR)

Total RNA was extracted from SCAP using TRIzol reagent (TaKaRa, Tokyo, Japan) according to the manufacturer’s protocol. The concentration and purity of RNA were quantified using UV spectroscopy. The Genomic DNA was excluded using gDNA Eraser in PrimeScript™ RT reagent Kit (TaKaRa, Tokyo, Japan) at 42 °C for 2 min, then cDNA was synthesized with 1 μg of total RNA. Real-time polymerase chain reaction (PCR) assays were performed on triplicate samples using SYBR Premix Ex TaqTM II (Takara, Tokyo, Japan) in a Roche 480 Light Cycler (Roche, Mannheim, Germany). The cycling conditions consisted of incubating at 95 °C for 30 s, 40 cycles of 95 °C for 5 s and 60 °C for 30 s. All target genes were normalized to the control endogenous glyceraldehyde-3-phosphate dehydrogenase (*GAPDH*; Genscript, Nanjing, China). Information about the primer sequences are listed in Table 1.

**Table 1:** Primer sequences used in qPCR

Gene	Primer sequences sense/anti-sense
GAPDH	5′- GCACCGTCAAGGCTGAGAAC-3′
	5′- TGGTGAAGACGCCAGTGGA-3′
BSP	5′- CTGGCACAGGGTATACAGGGTTAG −3′
	5′- GCCTCTGTGCTGTTGGTACTGGT −3′
Runx2	5′- TCCACACCATTAGGGACCATC-3′
	5′- TGCTAATGCTTCGTGTTTCCA-3′
DSPP	5′- GCATTTGGGCAGTAGCATGG-3′
	5′-CTGACACATTTGATCTTGCTAGGAG-3′
OCN	5′-AGGGCAGCGAGGTAGTGAAG-3’
	5′-CTCCTGAAAGCCGATGTGGT-3’

*GAPDH* glyceraldehyde-3-phosphate dehydrogenase, *BSP*, bone sialoprotein, *RUNX2* runt-related transcription factor 2, *DSPP* dentin sialophosphoprotein, *OCN* osteo-calcin

### Western blotting

The total protein was extracted from SCAP using RIPA lysis buffer (Jingcai Biotechnology, Xi’an, China) with 1 mM phenylmethylsulfonylfluoride (PSFM) (BeyotimeSC, Shanghai, China). The protein concentrations were determined by BCA protein assay reagent (Keygente, Nanjing, China). Equal amount of protein samples were loaded and separated by 10% sodium dodecyl sulfate-polyacrylamide gel electrophoresis (SDS-PAGE) and transferred onto polyvinylidene fluoride (PVDF) membrane. After blocking with 5% (w/v) nonfat dried milk at room temperature for 1 h, the membranes were incubated at 4 °C overnight with primary antibodies against DSPP, OCN (1:400; Santa Cruz Biotech, Santa Cruz, CA, USA), BSP, RUNX2 (1:400; Boster, Wuhan, China) and GAPDH (1:10000). The membranes were washed in TBST for three times, then they were incubated with the appropriate horseradish peroxidase conjugated secondary antibodies (1:100000; Boster, Wuhan, China) at room temperature for 1 h. The membranes were then visualized using enhanced chemiluminescence (ECL) (Merck Millipore, Billerica, MA, USA) and exposed to Kodak X-ray films.

To make sure whether MAPK signaling pathways were involved in the process, SCAP were treated with 0.2 mg/ml MTA, after 0, 5, 15, 30, 60, 120 min, the expression of total and phosphorylated protein (ERK/p-ERK, p38/p-p38, JNK/ p-JNK) (Merck Millipore, Billerica, MA, USA) were tested via Western blot with the same methods as above. In addition, cells were treated with MTA (0.2 mg/ml) for 12 h and MAPKs inhibitors (U0126, inhibitor of ERK, 20 μM; SB203580, inhibitor of p38, 20 μM) for 1 h, the experimental group design is shown in Table 2, then the ALP activity was analyzed on 3 and 5 d, the mRNA and protein expression were detected by qPCR and Western blot on 5 d.

**Table 2:** Experimental Group Design

Control	0 mg/ml MTA + 10% FBS α-MEM
MTA	0.2 mg/ml MTA + 10% FBS α-MEM
MTA + SB203580	0.2 mg/ml MTA + 10% FBS α-MEM + SB203580(20 μM)
MTA + U0126	0.2 mg/ml MTA + 10% FBS α-MEM + U0126(20 μM)

SB203580, inhibitor of p38; U0126, inhibitor of ERK

### Statistical analysis

The experiments were performed independently for each donor and the results were similar. The data were presented as mean ± standard deviation (SD). Comparisons between the experimental groups and control groups were performed using a two-tailed t-test or one-way ANOVA for experiments with more than two subgroups. Significance levels were set at *p < 0.05 and **p < 0.01. Statistical analysis was performed using GraphPad Prism 6.0.

## Results

### The characteristics of SCAP

**Fig. 1:**
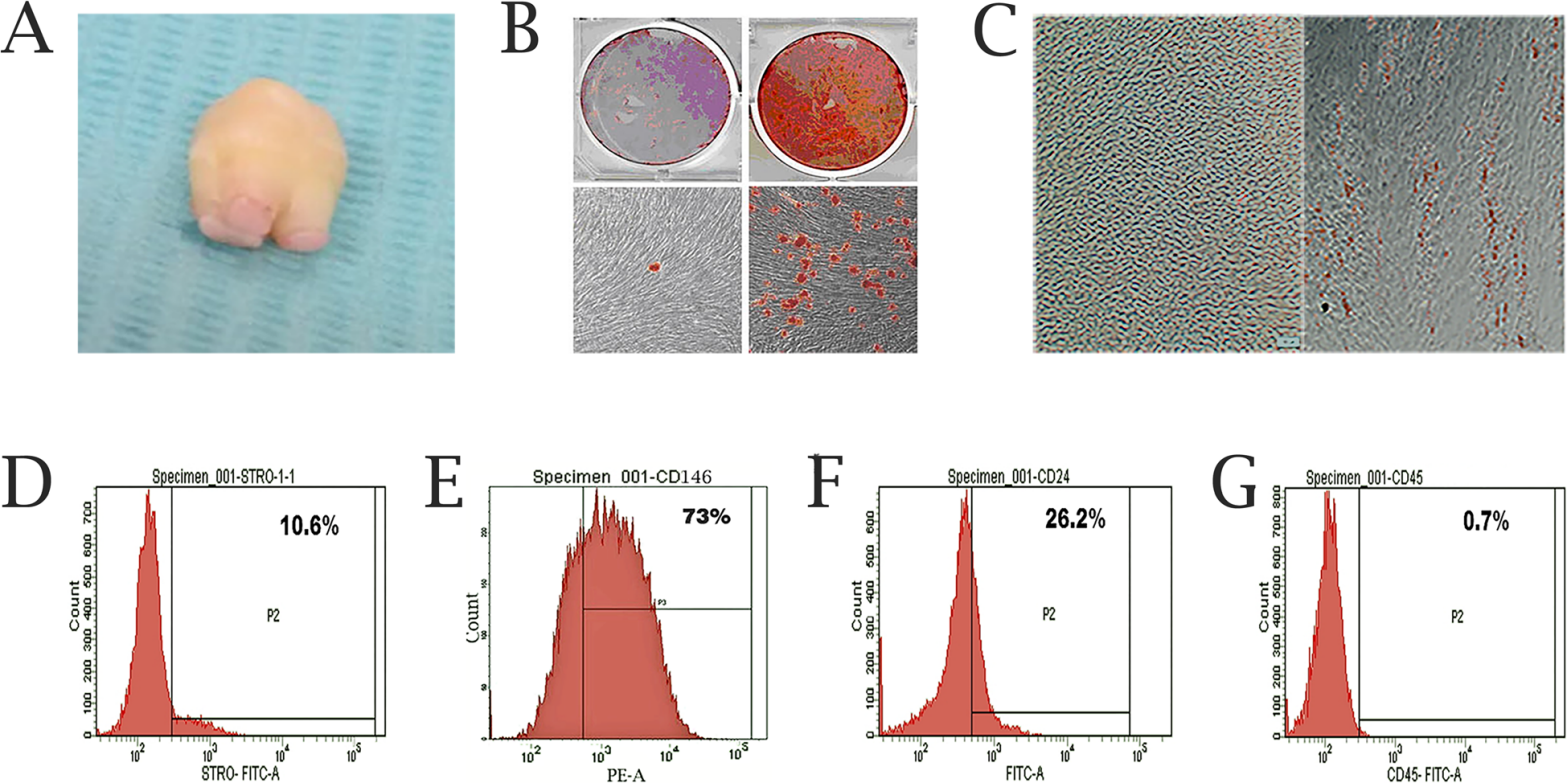
Characterizations of SCAP. **a** The apical papilla tissue from immature permanent teeth. **b** Mineralized nodules were observed after mineralization culture for 21 d. **c** Lipid droplets were observed after adipogenic induction for 4 wk. **d-g** Flow cytometric analyses of cell surface marker expression: STRO-1(10.6%), CD146 (73%), CD24 (26.2%), and CD45 (0.7%)

The SCAP were isolated from apical papilla located at the root apex of immature permanent teeth (Fig. 1a). Flow cytometric analysis indicated that SCAP express the MSC markers STRO-1, CD146 and SCAP specific phenotype CD24. The hematopoietic maker CD45 was expressed at low level (Fig. 1d, e, f and g). Alizarin Red staining showed that mineralized nodules were formed in SCAP cultured with mineralization medium (Fig. 1b). Lipid droplets were apparent after 4 wk. of adipogenic differentiation by Oil Red O staining (Fig. 1c). The above indicates that SCAP originate from MSCs rather than hematopoietic precursors.

### The effects of MTA on SCAP proliferations

**Fig. 2:**
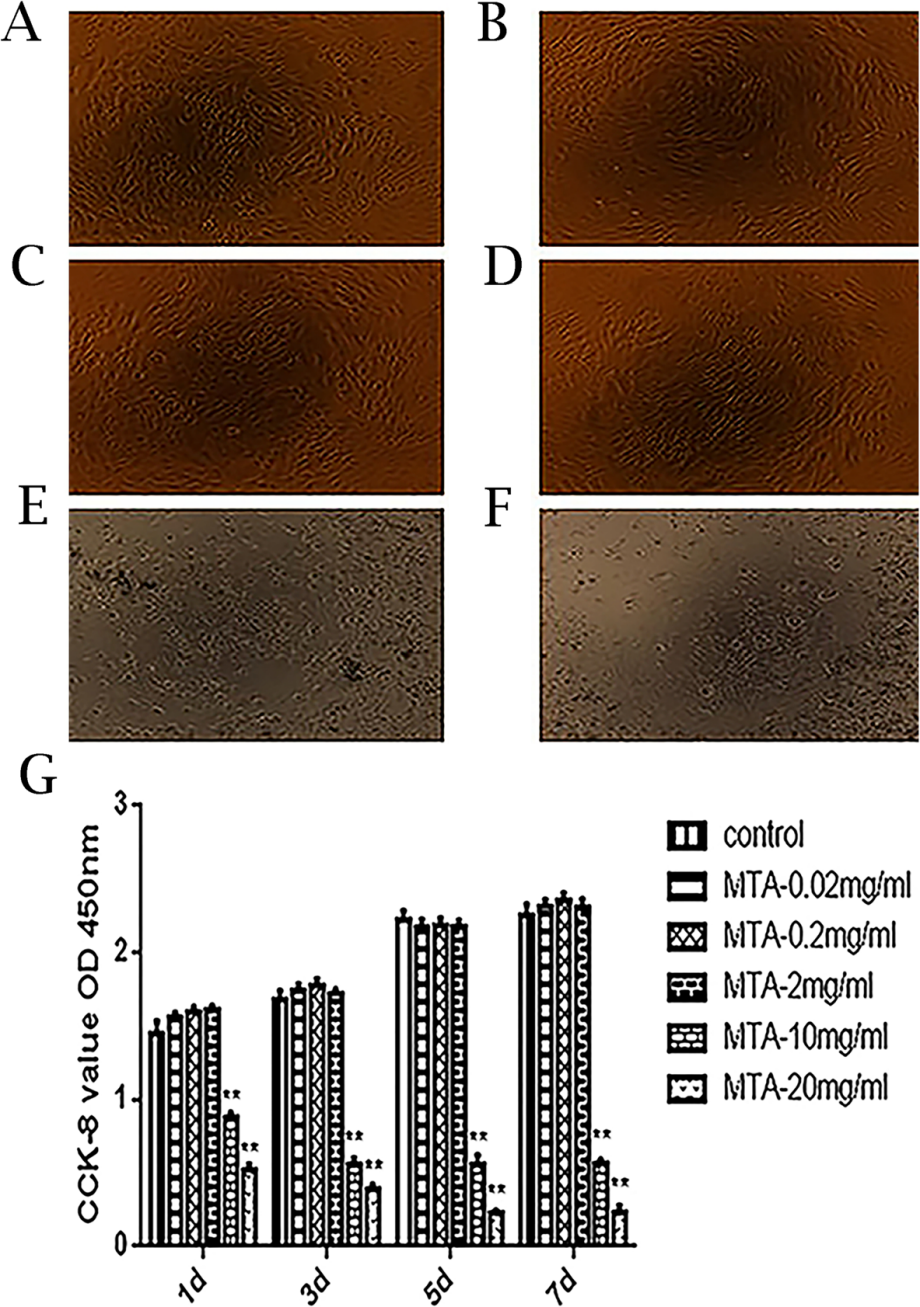
Effects of different MTA concentrations on the proliferation of SCAP proliferations. **a-f** The morphology of SCAP at different concentrations (0, 0.02, 0.2, 2, 10 and 20 mg/ml). **g** The proliferation of SCAP was analyzed using CCK-8 at 1, 3, 5 and 7 d (**p < 0.0001 at 10 and 20 mg/ml versus control)

As shown in Fig. 2, MTA at 10 mg/ml and 20 mg/ml inhibited SCAP proliferation (*p*<0.05), and we hardly observed normal morphological cells (Fig. 2e, f). We found that higher concentrations of MTA were cytotoxic to the cells (Fig. 2g). However, lower concentrations of MTA (0.02 mg/ml, 0.2 mg/ml and 2 mg/ml) did not exhibit a prominent effect on SCAP proliferation (*p* > 0.05). Meanwhile, no significant difference of morphology was observed using light microscopes (Fig. 2a, b, c and d).

### The effects of MTA on osteo/odontogenic differentiation of SCAP

**Fig. 3:**
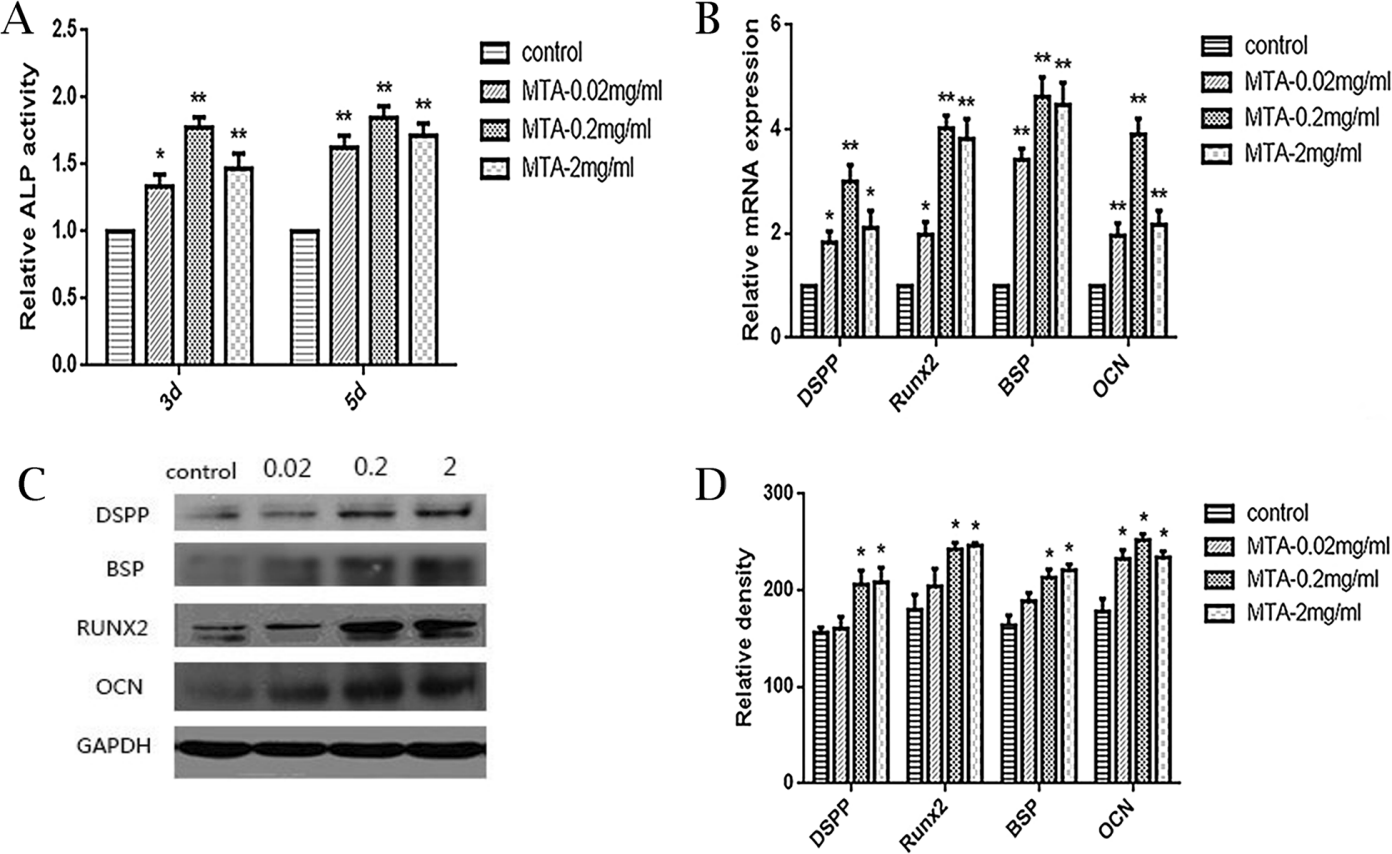
The effects of different MTA concentrations on osteo/odontogenic SCAP differentiation. **a** Alkaline phosphatase (ALP) activity in groups with different MTA concentrations (0, 0.02, 0.2, 2 mg/ml) on 3 d and 5 d. **b** Relative mRNA expression of DSPP, RUNX2, BSP and OCN in each group at 5 d. GAPDH served as a reference gene. **c** Protein expression of DSPP, RUNX2, BSP and OCN in different groups on 5 d. **d** Grayscale analysis of c. (*p < 0.05, **p < 0.01)

The ALP activity was higher in the MTA groups than control group on both days 3 and 5, and the 0.2 mg/ml MTA group exhibited the most significant differences (*p*<0.01) (Fig. 3a). The qPCR assays showed that mRNA expression of osteo/odontogenic genes (*DSPP*, *RUNX2*, *BSP*, and *OCN*) were up-regulated in the MTA groups compared with the control group on day 5 (*p*<0.05), while the highest expression were observed in 0.2 mg/ml and 2 mg/ml group (Fig. 3b). Western blotting shows that The proteins expression of DSPP, RUNX2, BSP, OCN were remarkably greater after the MTA treatment at 0.2 mg/ml and 2 mg/ml compared with the control group (Fig. 3c, Additional file 1: Figure S1 and Fig. 3d).

### MTA-activated MAPK signaling pathway in SCAP

**Fig. 4:**
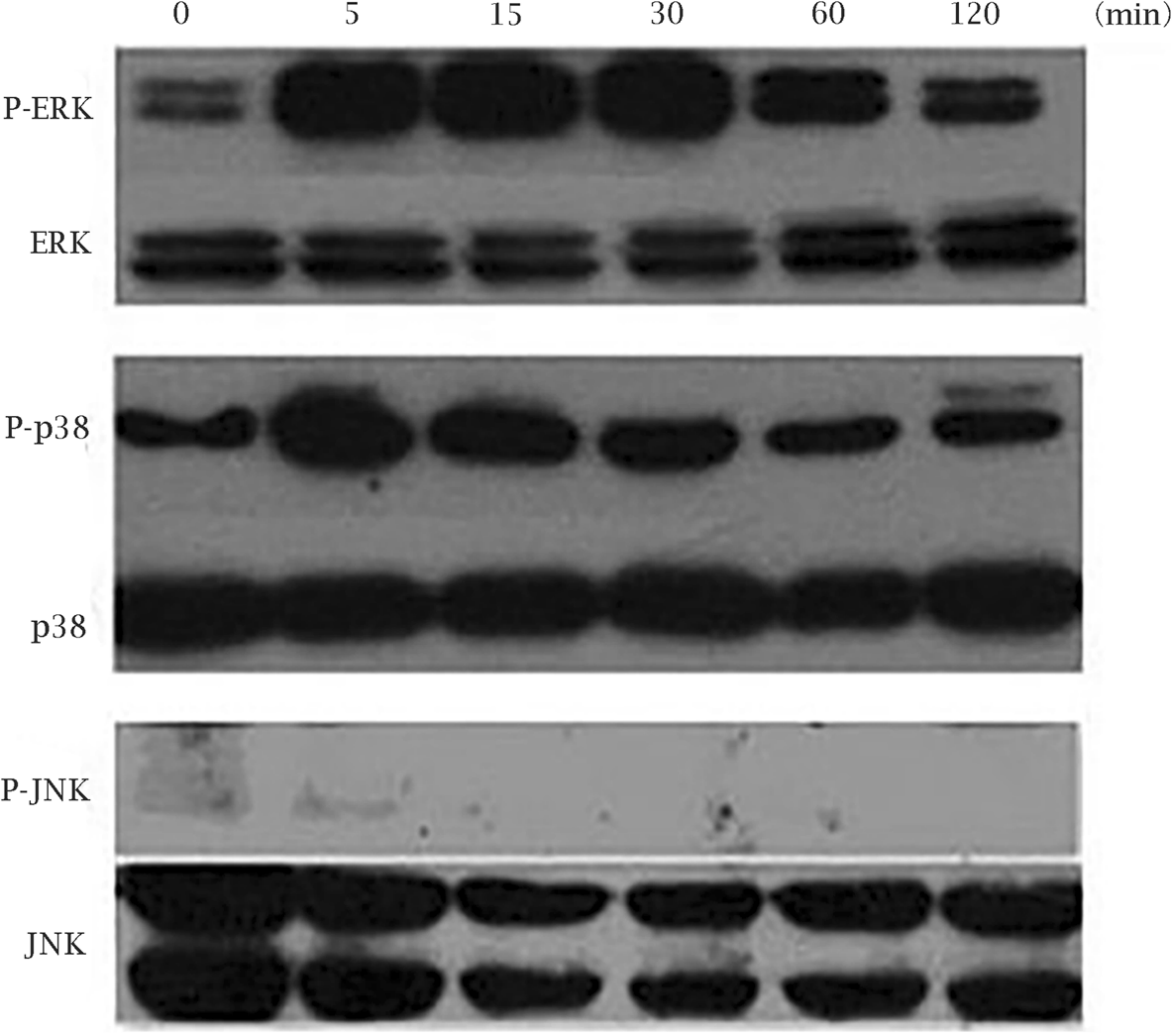
MTA-mediated MAPK signaling pathways in SCAP The expression of P-ERK, ERK, P-p38, p38, P-JNK, and JNK in MTA-treated SCAP at different time points (0, 5, 15, 30, 60 and 120 min)

To clarify the role of MAPKs signaling pathways on MTA-mediated osteo/odontogenic differentiation in SCAP, the total and phosphorylation protein of ERK, p38 and JNK were detected by Western blot. The results indicated that the p-p38 peaked at 5 min, p-ERK occurred within 5 min and reached a peak at 15 min. However, we detected a little expression of p-JNK which had no significant differences statistically (*p* > 0.05). Meanwhile, the gross level of ERK, p38 and JNK protein didn’t show obvious changes (Fig. 4 and Additional file 2: Figure S2).

### The effects of p38 and ERK inhibitors on MAPK signaling pathways in SCAP

**Fig. 5:**
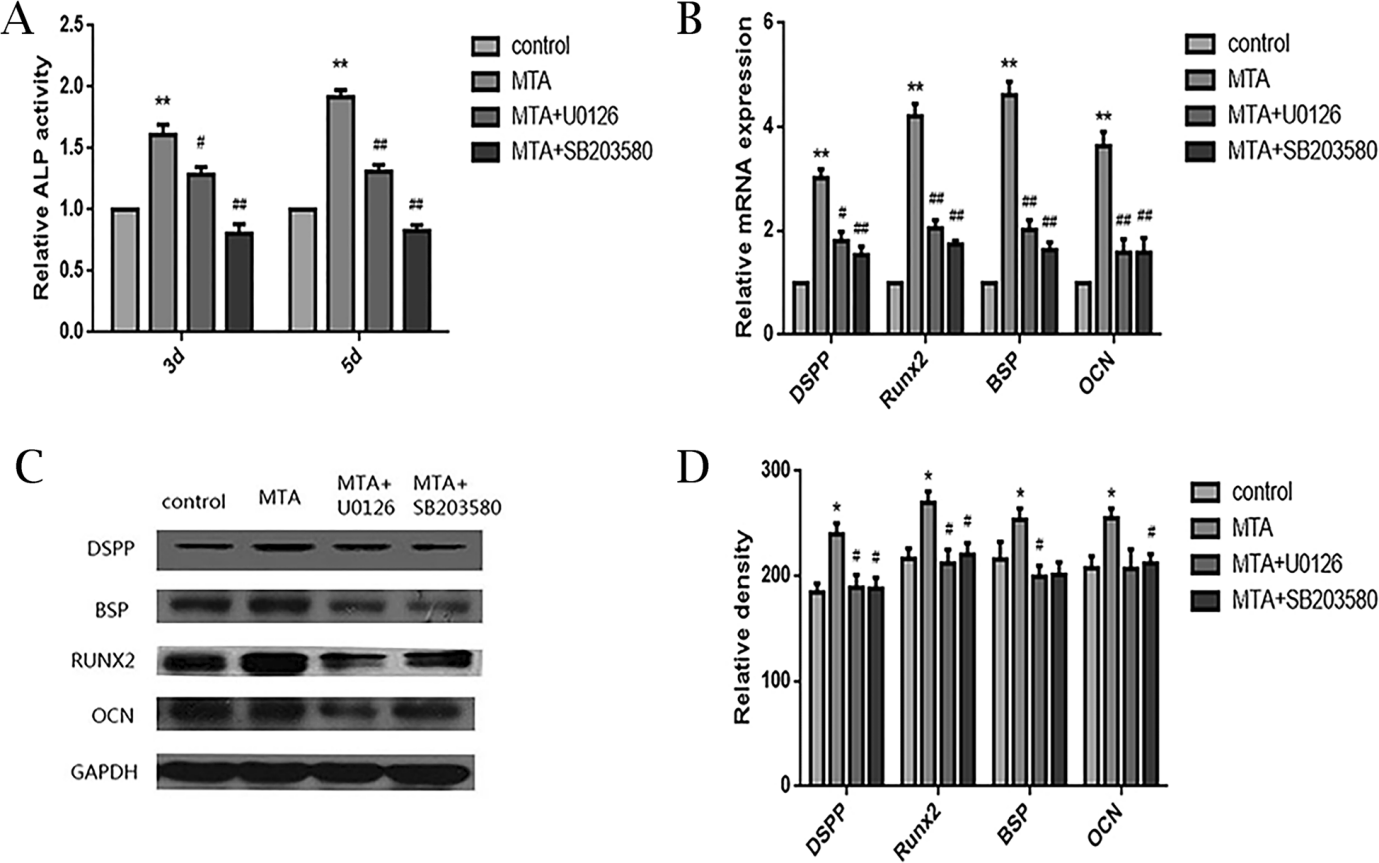
The effects of p38 and ERK inhibitors on MTA-mediated osteo/odontogenic differentiation in SCAP, **a** Alkaline phosphatase (ALP) activity in different groups on 3 d and 5d. **b** Relative mRNA expression of DSPP, RUNX2, BSP and OCN at 5 d. GAPDH served as a housekeeping gene. **c** Protein expression of DSPP, RUNX2, BSP and OCN in different groups at 5 d. **d** Grayscale analysis of c. (*p < 0.05, **p < 0.01, significantly different vs. control. ^#^*p* < 0.05, ^##^*p* < 0.01, significantly different vs. MTA-treated group)

To further verified the role of MAPK signaling pathways, the p38 and ERK inhibitors (SB203580 and U0126) were added to the culture system. As shown in Fig. 5, ALP activity decreased after treated with SB203580 and U0126, compared to the MTA group on days 3 and 5 (Fig. 5a). It revealed that these inhibitors decreased mRNA expression of *RUNX2*, *DSPP*, *OCN*, and *BSP* via qPCR assay (Fig. 5b). Western blot demonstrated that the protein expression decreased at days 5 after exposure to the inhibitors (Fig. 5c, Additional file 3: Figure S3 and Fig. 5d). Based on the above, the p38 and ERK inhibitors decreased ALP activity and inhibited mRNA and protein expression of RUNX2, DSPP, OCN, and BSP.

## Discussion

With good sealing ability and biocompatibility, MTA has been widely used in endodontic treatment and usually obtained admirable results. As an effective pulp-capping material, MTA facilitates reparative dentine formation at the exposed pulp [17, 18]. In addition, previous study has shown that MTA promoted the odontogenic differentiation of pulp cells in vitro [22]. During apexification, MTA could directly interact with the apical papilla tissue and enhance hard tissue formation in immature permanent teeth [21]. The SCAP are Mesenchymal stem cells originated from apical papilla tissue and participate in the root dentin formation during tooth development [28]. However, the effect of MTA on Proliferation and osteo/odontogenic differentiation in SCAP and related mechanism remain unclear.

In previous study, Hakki et al. prepared MTA supernatant fluid and investigated the effect of MTA at different doses on cementoblasts. The results indicated that higher concentrations of MTA (20 mg/ml) was cytotoxicity to cementoblasts, and lower concentrations (0.02 mg/ml and 0.002 mg/ml) enhanced cell survival [29]. The same method was used to generate an MTA medium in the present study. The CCK-8 assay suggested that 10 and 20 mg/ml MTA significantly inhibited proliferation on account of its toxic effect to cells, but there were no apparent effect at lower concentrations (0.02, 0.2 and 2 mg/ml). Thus, lower concentrations of MTA were used in the following experiments.

The SCAP could be induced to differentiate into osteoblasts and odontoblasts which express high level of DSPP and other mineral markers in vitro [30, 31]. The ALP activity is an early indicator of osteo/odontogenic differentiation and is closely related to mineralization [32]. This study demonstrated that ALP activity rose with the MTA treatment, and the most notable change was found in 0.2 mg/ml group. The specific marker for odontogenic differentiation, *DSPP*, is in the late stage and plays a vital role in dentinogenesis [30, 33, 34]. After treated with different concentrations of MTA for 5 d, the mRNA expression of *DSPP* was up-regulated and the most significant change was observed at 0.2 mg/ml group. Meanwhile, the protein expression of DSPP was remarkably increased in the 0.2 mg/ml and 2 mg/ml groups. Bone sialoprotein (*BSP*) is mainly secreted by osteoblasts and a crucial indicator of matrix deposition and mineralization [35]. Runt-related transcription factor 2 (*Runx2*) is indispensable for osteo/odontoblast differentiation and regulates numerous bone- and tooth-related gene expressions [36]. Osteocalcin (*OCN*) which synthesised and secreted by mature osteoblasts and osteocytes is widely used as a reliable indicator for the activity of bone formation [37]. In our study, the mRNA and protein expressions of these indicators increased in 0.2 mg/ml and 2 mg/ml groups according to qPCR and Western blot. Thus, we found that the osteo/odontogenic markers, both early-stage (*ALP, Runx2*) and late-stage (*DSPP, OCN*) were up-regulated at proper concentration, so we speculated that MTA could contribute to the osteo/odontogenic differentiation of SCAP. Based on the above data, 0.2 mg/ml and 2 mg/ml MTA are suitable for inducing osteo/odontogenic differentiation of SCAP. Therefore, 0.2 mg/ml was selected as the optical MTA concentration and used in the following study of MAPK signaling pathways.

As an ancient set of serine/threonine kinases, Mitogen-activated protein kinases (MAPKs) mediate the response to plenty of stimuli. Recent reports has investigated that MAPKs are vital signal transducers for bone formation regulation [38, 39]. There are three well-known groups of MAPKs containing ERK, p38, and JNK. ERK has two subtypes, ERK1 (MAPK3) and ERK2 (MAPK1), both of them are expressed in osteoblasts [24, 38]. Other studies have shown that p38 MAPK pathway can promote osteoblastic differentiation [39, 40]. However, the JNK MAPK pathway on osteo/odontogenic differentiation hasn’t been widely investigated so far. Zhao et al. reported that MTA can activate MAPK signaling pathways to regulate the osteo/odontogenic differentiation of dental pulp cells [27]. To clarify the effect of MTA on MAPK signaling pathways in osteo/odontogenic differentiation of SCAP, we detected the ERK/p-ERK, p-38/p-p38, JNK/p-JNK using Western blot. P-p38 and p-ERK increased after treated with MTA, which indicated that the p38 and ERK signaling pathways may participate in MTA-mediated osteo/odontogenic differentiation. However, the level of p-JNK did not change significantly, suggesting that JNK MAPK pathway was not activated by MTA in SCAP.

To further verify the roles of the ERK and p38 pathways in osteo/odontogenic differentiation of SCAP, U0126 (ERK inhibitor) and SB203580 (p38 inhibitor) were added in this study. Subsequently, ALP activity, mRNA and protein expression of mineralization indicators decreased after treated by SB203580 and U0126. The results indicated that MTA could activate the p38 and ERK signaling pathways in osteo/odontogenic differentiation of SCAP.

This study provided a new insight into the role of MTA in SCAP differentiation. Further studies are necessary to elucidate the other mechanisms involved in osteo/odontogenic differentiation of SCAP, which would benefit the application of MTA in endodontic treatment.

## Conclusions

The study demonstrated that MTA does not influence proliferation of SCAP in lower concentrations, While, in higher concentrations it is cytotoxic. MTA could enhance osteo/odontogenic differentiation of SCAP at appropriate concentration by activating p38 and ERK signaling pathways. It is speculated that the use of MTA may promote the formation of root dentine in apex which provides a new idea for the clinical application of MTA and the treatment of endodontic diseases.

## Supplementary information

**Additional file 1: Figure S1.** Original gel images of Fig. 3c.

**Additional file 2: Figure S2.** Original gel images of Fig. 4.

**Additional file 3: Figure S3.** Original gel images of Fig. 5c.

## Data Availability

The dataset used and/or analyzed during the current study available from the corresponding author on reasonable request.

## Abbreviations

BSA: Bovine serum albumin
BSP: Bone sialoprotein
CCK-8: Cell counting kit-8
DSPP: Dentin sialophosphoprotein
ERKs: Extracellular signal-regulated kinases
FBS: Fetal bovine serum
FCM: Flow cytometric
GAPDH: Glyceraldehyde-3-phosphate dehydrogenase
JNKs: c-Jun N-terminal kinases
MAPKs: Mitogen-activated protein kinases
MTA: Mineral trioxide aggregate
OCN: Osteocalcin
PDLSCs: Periodontal ligament stem cells
Runx2: Runt-related transcription factor 2
SCAP: Stem cells from apical papilla
α-MEM: Alpha-Modification of Eagle’s Medium
