# Supplementary figures and images for: Effects of ERK/p38 MAPKs signaling pathways on MTA-mediated osteo/odontogenic differentiation of stem cells from apical papilla: a vitro study
Source: BMC Oral Health. 2020 Feb 12;20:50. doi: 10.1186/s12903-020-1016-x (PMC7017546; doi:10.1186/s12903-020-1016-x)

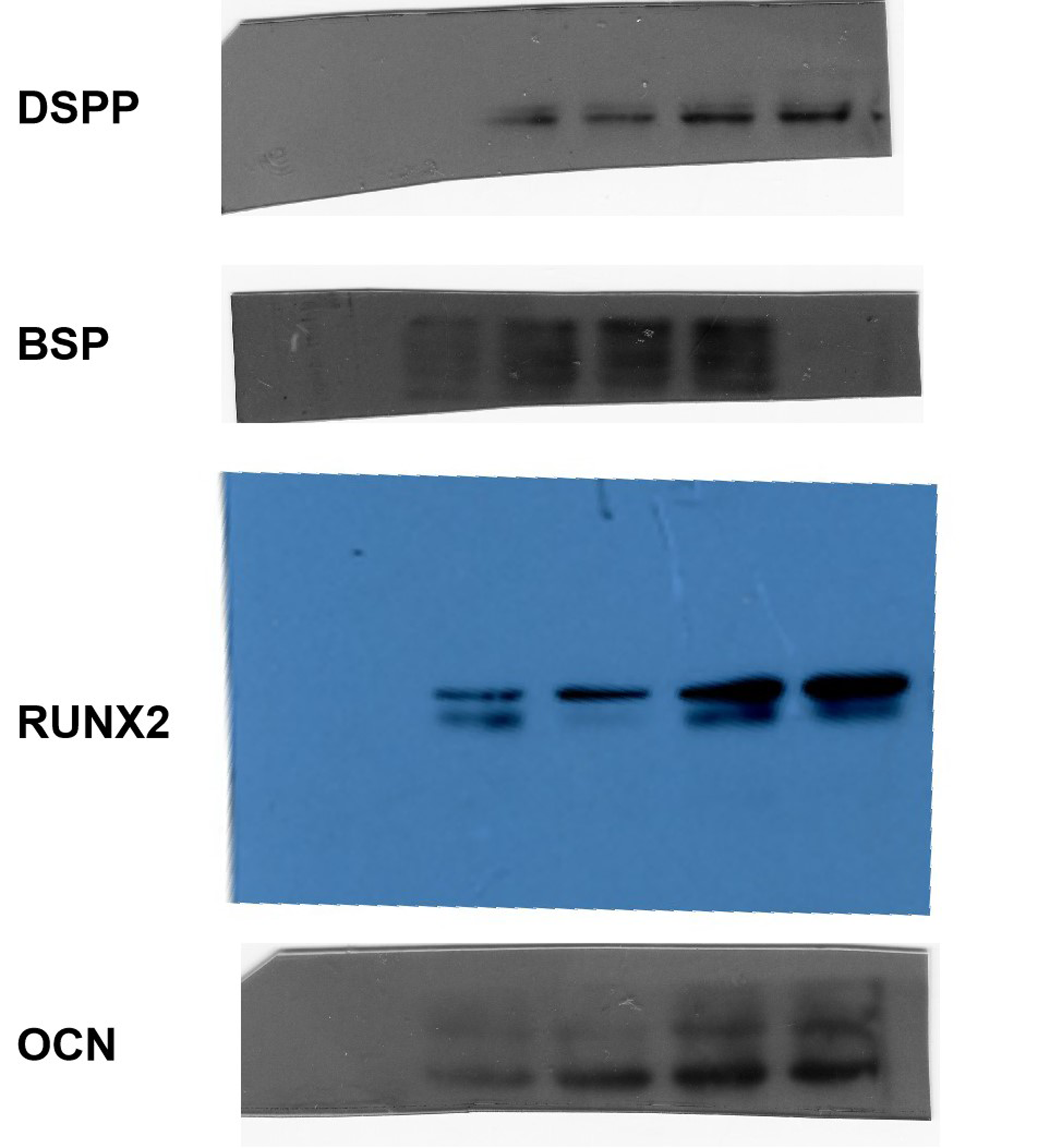

Supplement: Supplementary file 1 — Additional file 1: Figure S1. Original gel images of Fig. 3c. [file 12903_2020_1016_MOESM1_ESM.tif]

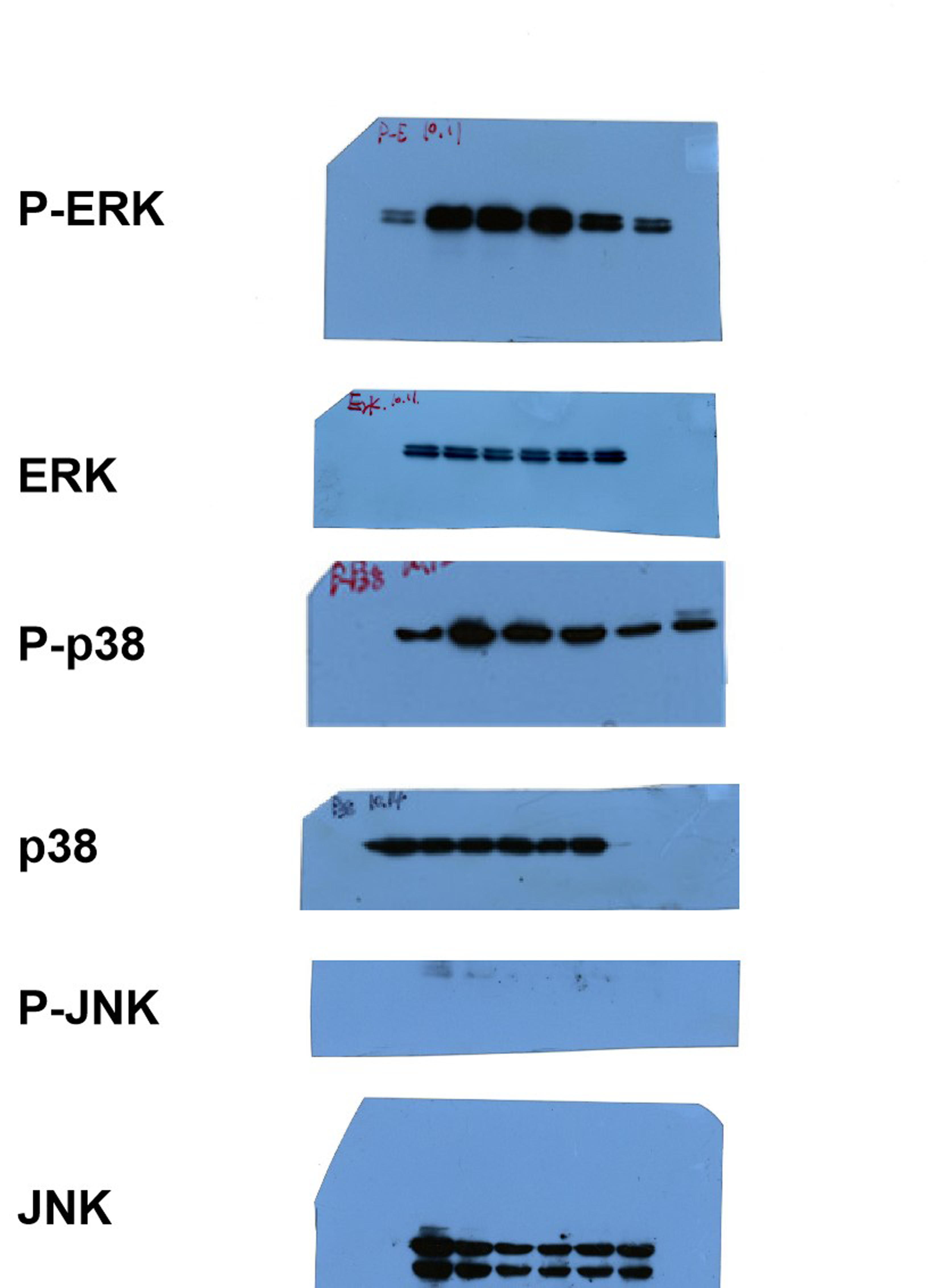

Supplement: Supplementary file 2 — Additional file 2: Figure S2. Original gel images of Fig. 4. [file 12903_2020_1016_MOESM2_ESM.tif]

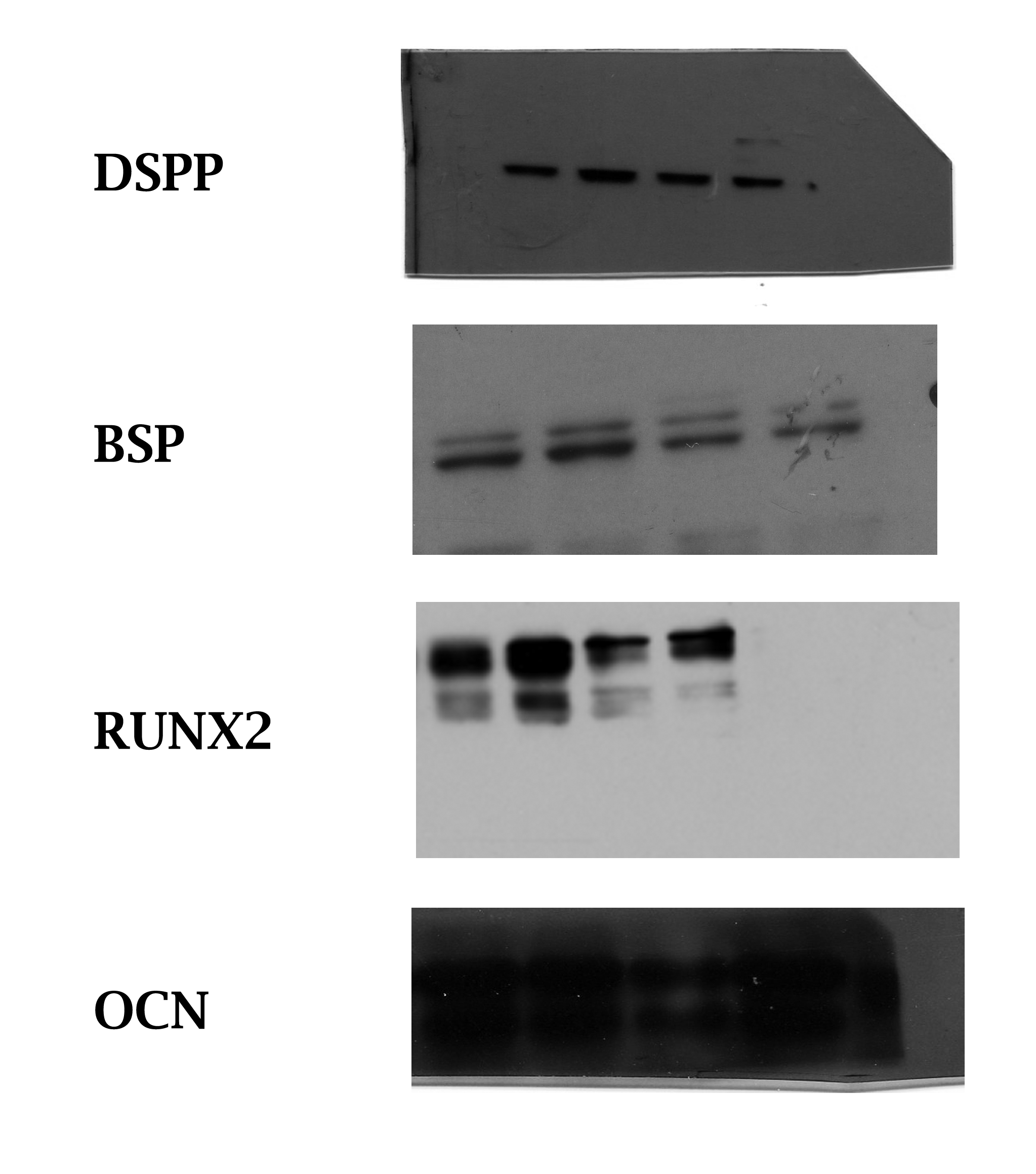

Supplement: Supplementary file 3 — Additional file 3: Figure S3. Original gel images of Fig. 5c. [file 12903_2020_1016_MOESM3_ESM.tif]
